# Long-term efavirenz pharmacokinetics is comparable between Tanzanian HIV and HIV/Tuberculosis patients with the same CYP2B6*6 genotype

**DOI:** 10.1038/s41598-018-34674-3

**Published:** 2018-11-05

**Authors:** Eliford Ngaimisi Kitabi, Omary Mashiku Sylivester Minzi, Sabina Mugusi, Philip Sasi, Mohamed Janabi, Ferdinand Mugusi, Leif Bertilsson, Jürgen Burhenne, Eleni Aklillu

**Affiliations:** 1 Department of Clinical Pharmacy and Pharmacology, School of Pharmacy, Muhimbili University of Health and Allied Sciences, Dar es Salaam, Tanzania; 2 Division of Clinical Pharmacology, Department of Laboratory Medicine, Karolinska Institutet at Karolinska University Hospital, Huddinge, Stockholm, Sweden; 3 Department of Clinical Pharmacology, School of Medicine, Muhimbili University of Health and Allied Sciences, Dar es Salaam, Tanzania; 4 Department of Clinical Pharmacology and Pharmacoepidemiology, University of Heidelberg, Heidelberg, Germany; 5 Department of Internal Medicine, Muhimbili University of Health and Allied Sciences, Dar es Salaam, Tanzania

## Abstract

The impact of anti-tuberculosis co-treatment on efavirenz (EFV) exposure is still uncertain as contradictory reports exist, and the relevance of CYP2B6*6 genetic polymorphism on efavirenz clearance while on-and-off anti-tuberculosis co-treatment is not well investigated. We investigated the determinants of long-term efavirenz pharmacokinetics by enrolling HIV (n = 20) and HIV/Tuberculosis (n = 36) subjects undergoing efavirenz and efavirenz/rifampicin co-treatment respectively. Pharmacokinetic samplings were done 16 weeks after initiation of efavirenz-based anti-retroviral therapy and eight weeks after completion of rifampicin-based anti-tuberculosis treatment. Population pharmacokinetic modeling was used to characterize variabilities and covariates of efavirenz pharmacokinetic parameters. CYP2B6*6 genetic polymorphism but not rifampicin co-treatment was the statistically significant covariate. The estimated typical efavirenz clearance in the HIV only subjects with the CYP2B6*1/*1 genotype was 23.6 L/h/70 kg, while it was 38% and 69% lower in subjects with the CYP2B6*1/*6 and *6/*6 genotypes, respectively. Among subjects with the same *CYP2B6* genotypes, efavirenz clearances were comparable between HIV and HIV/Tuberculosis subjects. Typical efavirenz clearances before and after completion of anti-tuberculosis therapy were comparable. In conclusion, after 16 weeks of treatment, efavirenz clearance is comparable between HIV and HIV/Tuberculosis patients with the same *CYP2B6* genotype. *CYP2B6* genotyping but not anti-tuberculosis co-treatment should guide efavirenz dosing to optimize treatment outcomes.

## Introduction

Efavirenz (EFV), a potent non-nucleoside reverse transcriptase inhibitor (NNRTI), is part of the first-line anti-retroviral combination therapies (cART) for HIV/AIDS in resource-limited countries. Tanzania is one of the resource-limited countries, greatly affected by the HIV epidemic with prevalence of 5% among adults aged 15–64 years. The burden of tuberculosis (TB) in Tanzania is also rising particularly among people living with HIV; HIV/TB co-infection accounted for 34% of the newly diagnosed cases of TB in 2016. Recent treatment guidelines recommend EFV containing ART as the default first-line regimens both in HIV-infected adults and children above 3 years of age^1^. It is also the default first-line ART regimen for HIV/TB co-treatment^1–3^. The levels of EFV plasma exposure have been related to HIV treatment outcomes. In a study by Marzolin *et al*., EFV levels below 1 μg/mL were associated with virological failure while levels above 4 μg/ml were associated with neuropsychiatric symptoms^4^. Efavirenz exhibits prolonged autoinduction leading to sub-therapeutic plasma levels in some patients after long-term treatment^5^. Furthermore metabolic disorders including, maldistribution, hyperlipemia, glucose intolerance and insulin resistance is recognized as long-term cART associated toxicities^6^. Hence long-term efavirenz pharmacokinetics may have implication on susceptibility to metabolic disorders including cardiovascular diseases.

The challenge to EFV based HIV treatment is the uncertainty of its adequate dosage due to inter-individual variation in the extent of its autoinduction and metabolism^5,7–9^. EFV is metabolized mainly by cytochrome P450 2B6 (*CYP2B6*) and to a lesser extent by *CYP2A6*, *CYP3A4* and uridine 5′-diphospho-glucuronosyltransferase (UGT) enzymes^10^. All the enzymes involved in efavirenz disposition are genetically polymorphic, with the frequencies of variant alleles varying not only among whites, Asians and blacks but also within sub-Saharan Africans^11,12^. For instance, the frequency of the CYP2B6*6 (c. 516 G > T) variant allele ranged from 8% in Finns to 50% in West Africans, while the frequency of UGT2B7*2 ranged from 47% in English and Scottish subjects to 80% in Nigerian Yoruba subjects^11^. We reported extensive genetic variations in *CYP2B6*, *CYP3A5*, *CYP2A6*, *UGT2B7*, *ABCB1* and *SLCO1B1* among black Africans and other populations^13–15^. The defective SLCO1B1*5 (c.521 T > C) variant allele occurs at a higher frequency in Europeans (23%), and the frequency distribution varies greatly among black Africans (19% in Ethiopians versus 3.2% in Tanzanians)^16,17^. The CYP2A6*9 allele frequency ranges from 21.9% in Koreans to 10.4% in Ugandans to 2.8% in Ethiopians^18,19^. While CYP2B6*6 occurs at a higher frequency in Tanzanians (42%) than in Ethiopians (31%), UGT2B7*2 occurs at a higher frequency in Ethiopians (48%) than in Tanzanians (29%)^13^. Using 4β-hydroxycholesterol as an endogenous marker, higher CYP3A enzyme activity in Ethiopians^16^, but significantly lower enzyme activity in Tanzanians than in Swedes or Koreans has been reported^20^. These population pharmacogenetic differences confer inter-ethnic and inter-individual variations in enzyme expression, activity, and inducibility^13,21,22^. Efavirenz exhibits prolonged autoinduction of its own metabolism. The extent of induction is dependent on the *CYP2B6* genotype, being highest in individuals with a wild-type genotype (faster metabolizers)^5,7–9,21^. This creates uncertainty regarding the therapeutic equivalence of equal efavirenz dosage across different individuals with different genotypes.

Rifampicin (RIF), which is the backbone of the anti-tuberculosis regimen, is a known promiscuous inducer of several EFV-metabolizing enzymes, with induction being genotype dependent and achieved faster than EFV-only induction^23–25^. This increases uncertainties about the therapeutic equivalence of efavirenz dosages in different individuals undergoing HIV/TB co-treatment. The anti-tuberculosis regimen containing rifampicin has been shown to increase efavirenz clearance in subjects with a wildtype *CYP2B6* allele but decreases the clearance in those homozygous for defective variant alleles^26^. In one of our previous studies, we found that in subjects with wildtype *CYP2B6* genotype, efavirenz steady-state concentrations were comparable between subjects on EFV-only and RIF/EFV co-treatment^9^. However, we have also reported that RIF/EFV co-treatment causes higher induction of the CYP3A enzyme compared to EFV alone^7,21^. Furthermore, inconsistency exists on the impact of RIF/EFV co-treatment on EFV exposure when short-term (4 weeks) EFV-RIF pharmacokinetic interaction studies are compared with long-term (≥12-week treatment) studies. Short-term studies have shown that RIF/EFV co-treatment results into 20–30% lower EFV exposure compared to EFV-only treatment, while in contrast, long-term studies have shown comparable and even higher EFV exposures with co-treatment^27–32^. It is therefore uncertain whether the efavirenz dosage when taken concomitantly with rifampicin containing anti-TB is bioequivalent to when it is taken in an ART-only regimen. The overall impact of HIV/TB co-treatment on EFV pharmacokinetics needs to be further investigated.

Previously we have conducted large multinational prospective cohort studies investigating the relevance of pharmacogenetic variations for efavirenz plasma exposure and treatment outcome in different African populations including Tanzanians. We reported the association of CYP2B6*6 genotype with cART induced liver toxicity in Ethiopians^33,34^ and Tanzanian patients^35^, and with neuropsychiatric toxicity in Ugandan^19^ and Tanzanian patients^36^, and with CD4 recovery among Ethiopian patients^8^. Investigation of long-term efavirenz pharmacokinetics with and without rifampicin co-treatment among Ethiopian subjects has indicated the independence of EFV pharmacokinetics on RIF/EFV co-treatment even after adjusting for *CYP2B6* polymorphisms^31^. However, we reported previously that the pharmacogenetics and pharmacokinetics of EFV display significant differences between Ethiopians and Tanzanians^13,16^. Considering the impact of genetic and environmental factors on the metabolic activity of enzymes, it is important to study efavirenz pharmacokinetics in different populations.

We aimed to investigate the determinants of the efavirenz pharmacokinetic parameters after long-term ART with and without rifampicin co-treatment. In particular, we sought to assess the influence of RIF/EFV co-treatment, genetic polymorphisms in efavirenz-metabolizing enzymes, and demographic characteristics on EFV pharmacokinetics among Tanzanian HIV patients with or without tuberculosis co-infection.

## Material and Methods

### Study design and patient population

The study was designed to enrol stable HIV patients on EFV-containing ART regimens who were undergoing HIV-only or HIV/TB co-treatment. Therefore, patients who were participating in a controlled observational study entitled “optimization of HIV/TB co-treatment in Africa” and who gave informed consent were recruited to participate in this study. The cohort description and the main clinical features of the study participants have been reported previously^9^. Briefly, it was a treatment, non-randomized, open-label, active control, parallel assignment, and population steady-state pharmacokinetic and pharmacogenetic study in which treatment-naive HIV and HIV/TB patients (with CD4+ T helper cell counts < 200/mL) were recruited into arm 1 and arm 2, respectively. Arm 1 was initiated on an EFV-based ART regimen, while patients in arm 2 were first initiated on a 6-month RIF-based anti-TB regimen followed by initiation of EFV-based ART 4 weeks after anti-TB initiation. The oral ART regimen consisted of once daily 600 mg of EFV, twice daily 150 mg of Lamivudine (3TC) and either twice-daily doses of 300 mg of zidovudine (AZT), 30/40 mg of stavudine (D4T) or 300 of tenofovir (TDF). The anti-TB regimen consisted of oral daily doses of 10 mg/kg (maximum 600 mg) of RIF, 5 mg/kg (maximum 300 mg) of isoniazid, 25 mg/kg (maximum 1600 mg) of pyrazinamide, and 15 mg/kg (maximum 1100 mg) of ethambutol as recommended in the Tanzania Manual for the Management of Tuberculosis and Leprosy^37^. Except for RIF and isoniazid none of the other co-administered drugs have known pharmacokinetic interaction with EFV. The study protocol received ethics approval from the Muhimbili University of Health and Allied Sciences and the Karolinska Institute ethical review committees.

In the current sub-study, subjects in arm 1 were recruited for intensive blood sampling at week 16 of EFV treatment. Subjects in arm 2 were recruited for intensive blood sampling during HIV/TB co-treatment on two occasions. Samples were first taken just before completion of the anti-TB therapy (at least week 20 of RIF and 16 weeks of antiretroviral co-treatment ART). A second plasma sample was taken 8 weeks after completion of the anti-TB co-treatment (occasion 2, at least 32 weeks of RIF initiation). Considering a 20% within subject variability in EFV AUC a sample size of 20 subjects was required to detect a 20% difference in efavirenz clearance between arm1 and arm2 at an 80% power and type 1 error rate of 5%^38^. However, more subjects were recruited in arm 2 such that at least 20 subjects could be available for the second PK sampling. The schematic representation of the timelines of treatment initiation and PK sampling for the two arms is shown in the Supplementary Material, Figure S1.

Genotype information during the 16^th^ clinical week and demographic characteristics were obtained from the main study database. Methods for genomic DNA extraction and genotyping of CYP2B6*6, CYP3A5*3, *5 and *7, UGT2B7*2, *ABCB1 c*.*3435 C > T*, *ABCB1 c*.*3842 A > G*, and SLCO1B1*1b and *5 were as reported previously^17,35^.

### Blood sampling and quantification of plasma efavirenz concentration

Subjects selected for this pharmacokinetic sub-study were admitted for 24 hours at Muhimbili National Hospital (MNH). Efavirenz is dosed once daily and is generally administered in the evening owing to its central nervous system side-effects. During study enrolment, subjects were instructed to take their daily doses of efavirenz between 20:00–21:00 pm local time if possible. On the day of admission subjects were administered EFV 600 mg at about 20:00 pm. The subjects stayed overnight for intensive PK sampling and were discharged on the evening of the next day. The subjects contributed blood samples from both arms at approximately 0, 1, 3, 6, 12, 16, and 24 hours after taking a 600 mg EFV tablet orally. Blood samples were immediately centrifuged, and plasma was stored at −80 °C. Samples were shipped on dry ice to the Department of Clinical Pharmacology and Pharmacoepidemiology, University of Heidelberg, Germany. Liquid chromatography-tandem mass spectrometry (LC/MS/MS) was used for quantification of plasma efavirenz as described previously^5^. Efavirenz**-[**^**13**^**C]** was used as an internal standard for efavirenz. The lower limit of quantification was 10.0 ng/mL, and the calibration range was between 10.0–10,000 ng/mL. Linear regression with 1/x weighing resulted in correlation coefficients of r^2^ = 0.99. The accuracy and precision (within-batch and batch-to-batch) of the assay fulfilled all the recommendations of the FDA guidelines.

### Population pharmacokinetic analysis of efavirenz concentrations

NONMEM software (Version 7.3; http://www.iconplc.com/innovation/nonmem/) was used for modelling. Perl-speaks-NONMEM (PsN, v 4.4.0; https://uupharmacometrics.github.io/PsN/) was used to operate NONMEM and R (version 3.3.1; www.r-project.org) for graphical inspection of the results. Efavirenz disposition kinetics were assessed by evaluation of 1- and 2-compartment pharmacokinetic models. In both evaluations, the oral absorption of efavirenz was considered through first-order absorption kinetics with or without lag time to the absorption. Simultaneous zero- and first-order absorption kinetics were also tested. The models were parameterized in terms of oral clearance (CL/F), apparent volume of the central compartment (V/F), absorption rate constant (KA), inter-compartment clearance (Q/F, for 2 compartment model) and apparent peripheral volume of the distribution (VP/F, for 2 compartment model). Model fitting and estimation of the model parameters was done using the first-order conditional estimation method with interaction.

Since the population distribution of biological parameters resembles a log-normal distribution, individual subject parameter values were modelled as the product of the typical value of a parameter and the exponent of the random effect (equation (1)).

1 $${P}_{i}={P}_{TV}\times {e}^{{\eta }_{i}}$$

where $${\eta }_{i}\sim N(0,{\omega }^{2})\,and\,{\omega }^{2}=variance\,of\,the\,random\,effects\,parameters\,(\eta )$$

Inter-occasion variability of CL/F was considered for arm 2 subjects to account for any unexplainable between occasion differences in CL/F; individual clearance at occasion *j*was calculated as indicated in equation (2)

2 $$C{L}_{ij}=C{L}_{TV}\times {e}^{{\eta }_{i}+{\kappa }_{ij}}$$

where $${\kappa }_{ij}\sim N(0,{\omega }^{2})\,and\,{\omega }^{2}=variance\,of\,the\,random\,effects\,parameters\,(\kappa )$$.

Model building followed these steps. First, structural models without any of the variance parameters were used to fit the data, and the variance parameters were added sequentially to build stochastic models. A better structural or stochastic model was chosen based on the decrease in the NONMEM computed objective functions (OFV) and the goodness-of-fit assessed graphically. For hierarchical stochastic models, a decrease of more than 3.84 units in the OFVs was considered a statistically significant improvement to the model fit. Several models for unexplained (residual) variability in the observed efavirenz concentrations were tested, including additive, proportional and combined (proportional + additive) residual error models. Covariate model building was done in three steps. The first step involved visual inspection of plots of random effect parameters ($\eta \,(ETA)$) versus covariates (demographic, genotypes, clinical and laboratory characteristics). In this step, the CYP2B6*6 genotype was a predictor for CL/F both in arm 1 and arm 2 (ETA shrinkage for CL/F = 4.3%). CL/F versus the *ABCB1 c3842A* > *G* plot indicated lower clearance for 2 subjects (in arm 2) with a homozygous genotype in the mutated alleles compared to all other subjects. The CL/F versus CYP3A5*1 plot indicated unexpected higher CL/F for 4 subjects (in arm 1) with a homozygous genotype in the mutated alleles compared to all other subjects. CYP2B6*6 was also a predictor for V/F in both arms (ETA shrinkage for V/F = 15%). No covariate was a predictor for KA (ETA shrinkage for KA = 37%). Smoking status was a predictor (in arm 1), while sex (in arm 1) and the type of anti-retroviral combination (in arm 2) indicated some trend to predict Q/F (ETA shrinkage for Q/F = 29%). However, the observed relationships for V/F and Q/F have no physiological explanation and therefore were not included in the covariate modelling. In the second step, a generalized additive model (GAM) of the CL/F versus treatment arm, CYP2B6*6, CYP3A5*1, and *ABCB1 c3842A* > *G* was evaluated. In this model, only the *CYP2B6* genotype was a significant predictor of clearance. Therefore, because of the small number of subjects and the lack of statistical significance in the GAM analysis, CYP3A5*1 and *ABCB1 c3842A* > *G* were not included in the last step of covariate modelling. ETA shrinkages for the base model were relatively low and supported covariate model development using the identified parameter covariate relationship. Lastly, a full covariate model building approach was used to develop the final model. In this approach, all covariates reported in the literature to influence efavirenz exposure and with physiological explanation were included. The included covariates were CYP2B6*6, sex and treatment arm (EFV with or without rifampicin). This approach was taken to control for the possible confounding effect of the other covariates on the effect of HIV/TB co-treatment on efavirenz pharmacokinetics, as the distribution of the covariates was different between the treatment arms^39^. The influence of weight on efavirenz clearance was modelled using a previous reported allometric scaling power model, with the allometric exponent fixed to 0.75^40^. Sex was included as a covariate on clearance as identified in other previous efavirenz population PK literature^41–43^. The effect of HIV/TB co-treatment on efavirenz clearance was assessed by determining the percent differences between arm 1 (week 16) and arm 2 (week 20) and between arm 2 (week 32) and arm 2 (week 20). These comparisons were stratified by the CYP2B6*6 genotypes as indicated in equation (3):

3 $$\begin{array}{ccc}C{L}_{TV(subPOP)} & = & C{L}_{11}\times {({{\Theta }}_{12})}^{B2}\times {({{\Theta }}_{13})}^{B3}\times {({({\theta }_{21\_1})}^{B1}\times {({\theta }_{21\_2})}^{B2}\times {({\theta }_{21\_3})}^{B3})}^{A2}\,\\  &  & \times \,{({({\varphi }_{22\_1})}^{B1}\times {({\varphi }_{22\_2})}^{B2}\times {({\varphi }_{22\_3})}^{B3})}^{O2}\times {(\frac{WT}{70})}^{0.75}\end{array}$$

where the subscript notations 11, 12, and 13 represent arm 1 subjects with CYP2B6*1/*1, *1/*6 and *6/*6 genotypes, respectively. The subscript notations 21_1, 21_2, and 21_3 represent arm 2 subjects at week 20 with CYP2B6*1/*1, *1/*6 and *6/*6 genotypes, respectively. Lastly, the 22_1, 22_2, and 22_3 notations represent arm 2 subjects at week 32 with CYP2B6*1/*1, *1/*6 and *6/*6 genotypes, respectively. The superscript notations, B1, B2, and B3 represent *CYP2B6* genotypes *1/*1, *1/*6 and *6/*6 and take the value of 1 for yes or 0 for no. The A2 and O2 notations represent arm 2 and occasion 2 for subjects in arm 2, respectively, and take the value of 1 for yes or 0 for no. The Θ, θ, and ∅ notations represent the fraction of clearances between the compared subpopulations.

An alternative approach to explore the effect of HIV/TB co-treatment on efavirenz clearance could have been to use a model to account for the time-dependent increase or decrease in efavirenz clearance after subjects completed TB treatment (at week 20). However, this approach was not considered because subjects contributed samples at 2 occasions only (week 20 and week 32). This could have prevented identification of appropriate induction model and/or robust estimation of its parameters.

Due to a large proportion of missing CYP2B6*6 genotype data (19 of 56 subjects had no genotype information), population mixture modelling (as implemented with NONMEM) was used to impute the missing genotype information (See NONMEM code in the Supplementary Material). A sensitivity analysis was performed to explore improvement in model fit after genotype imputation. In this sensitivity analysis, objective function value (OFV) of a model without imputation was compared to the final model in which missing *CYP2B6* genotype data were imputed. The model with mixture modelling (to impute genotype data) had 10 units lower OFV (per one additional parameter) compared to the model without imputation, thus indicating improvement in the model fit (See Supplementary Material Table S1).

Evaluation of the adequacy of the final model to describe the data was done by assessing the goodness-of-fit (GOF), a visual predictive check (VPC) and 95% non-parametric bootstrap confidence intervals of the model parameters. The GOF was assessed graphically by evaluation of the agreement between the observed and predicted plasma concentrations, the range of individually weighted residuals (IWRES), and the uniformity distribution of these residuals about zero across the range of the predicted concentrations and time after dose. Epsilon shrinkages for the base (EPS shrinkage = 12%) and final model (Supplementary Table S2) were relatively low and supported the adequacy of the diagnostic GOF plots for model evaluation. Visual predictive check was performed by overlaying the observed concentration time profiles over the simulated profiles. The final model was considered adequate if the 5^th^, median and 95^th^ percentile of the observed data were contained within the 95% confidence interval bands of the 5^th^, 50^th^ and 95^th^ percentile of the simulated data. The confidence intervals were the 2.5^th^ −97.5^th^ percentiles of the parameters estimated by fitting the final model to 200 datasets bootstrapped from the original dataset. Non-parametric bootstrap sampling was stratified on the treatment arms and the CYP2B6*6 genotype. The non-parametric bootstrap confidence intervals were obtained after correction for bias and skewness of the bootstrapped parameters using the bias correction and acceleration method^44^.

## Results

### Subjects characteristics

Most subjects were mid-age with a median (IQR) age of 42 years^36–50^ and median (IQR) weight of 48 kilograms^43–60^. At the time of PK sampling (Week 16), the study participants between the two arms had differences in age, weight, Karnofsky score, types of combination ART and sex distribution (Table 1). Before of the initiation of anti-TB or ART the study participants between the two arms were different in the prevalence of syphilis infection, smoking and alcohol use status, immunological status (CD4), and serum creatinine and albumin levels (Table 1). Albumin and creatinine concentrations were not assessed at week 16, 20 and 32.

**Table 1:** Subject characteristics observed at week 16 (for Arm 1) and week 20 (for Arm 2).

Characteristic	Level	Arm 1 (N = 20)	Arm 2 (N = 36)	p-value
SEX (%)	Female	15 (75.0)	13 (36.1)	0.012
	Male	5 (25.0)	23 (63.9)	
*CYP2B6* c.516 G > T(*6) (%)	CYP2B6*1/*1	7 (35.0)	11 (30.6)	0.292
	CYP2B6*1/*6	7 (35.0)	6 (16.7)	
	CYP2B6*6/*6	2 (10.0)	4 (11.1)	
	Missing	4 (20.0)	15 (41.7)	
ABCB1 c.3435 C > T (%)	CC	8 (40.0)	17 (47.2)	0.009
	CT	8 (40.0)	2 (5.6)	
	TT	0 (0.0)	2 (5.6)	
	Missing	4 (20.0)	15 (41.7)	
ABCB1 c.3842 A > G (%)	AA	10 (50.0)	7 (19.4)	0.076
	AG	6 (30.0)	12 (33.3)	
	GG	0 (0.0)	2 (5.6)	
	Missing	4 (20.0)	15 (41.7)	
^¥^Number of CYP3A5*1 allele (%)	*1/*1	5 (25.0)	9 (25.0)	0.351
	*1/*0	7 (35.0)	8 (22.2)	
	*0/*0	4 (20.0)	4 (11.1)	
	Missing	4 (20.0)	15 (41.7)	
UGT2B7*2 g. 372 G > A (%)	AA	0 (0.0)	3 (8.3)	0.088
	AG	6 (30.0)	10 (27.8)	
	GG	10 (50.0)	8 (22.2)	
	Missing	4 (20.0)	15 (41.7)	
^‡^SLCO1B1*1b and *5 (%)	*1a/*1b	3 (15.0)	2 (5.6)	0.169
	*1b/*15	3 (15.0)	1 (2.8)	
	*1b/*1b	9 (45.0)	18 (50.0)	
	Missing	5 (25.0)	15 (41.7)	
Type of combination ART (%)	D4T/3TC/EFAVIRENZ	2 (10.0)	20 (55.6)	0.004
	TNF/EMT/EFAVIRENZ	1 (5.0)	1 (2.8)	
	AZT/3TC/EFAVIRENZ	17 (85.0)	15 (41.7)	
Smoking status (%)	Used to smoke	1 (5.0)	13 (36.1)	0.024
	Never smoked	19 (95.0)	23 (63.9)	
Alcohol use status (%)	Take alcohol	11 (55.0)	4 (11.1)	0.001
	Never take alcohol	9 (45.0)	32 (88.9)	
Shingles virus infection status (%)	Suffered Shingles	3 (15.0)	3 (8.3)	0.747
	Never had Shingles	17 (85.0)	33 (91.7)	
Hepatitis B infection status (%)	Never had HBV	18 (90.0)	35 (97.2)	0.596
	Suffered HBV	2 (10.0)	1 (2.8)	
Syphilis infection status (%)	Never had Syphilis	15 (75.0)	35 (97.2)	0.034
	Had Syphilis	5 (25.0)	1 (2.8)	
Karnofsky score (%)	70	8 (40.0)	1 (2.8)	0.001
	80	1 (5.0)	8 (22.2)	
	90	1 (5.0)	9 (25.0)	
	100	10 (50.0)	18 (50.0)	
AIDS stage (%)	1	1 (5.0)	6 (16.7)	0.060
	2	15 (75.0)	14 (38.9)	
	3	4 (20.0)	13 (36.1)	
	4	0 (0.0)	3 (8.3)	
Weight status (%)	Underweight	11 (55.0)	10 (27.8)	0.120
	Normal weight	7 (35.0)	22 (61.1)	
	Obese	2 (10.0)	4 (11.1)	
Weight (kg, mean (sd))		45.50 (13.15)	55.14 (13.45)	0.012
Age (years, mean (sd))		45.84 (8.56)	39.77 (9.63)	0.022
Height (cm, mean (sd))		163.55 (7.08)	163.78 (7.81)	0.914
^₤^Baseline absolute CD4 count (unit, mean (sd))		68.25 (36.62)	105.97 (77.08)	0.045
^₤^Baseline serum creatinine concentration (mmol/L, mean (sd))		78.40 (11.46)	67.27 (24.01)	0.057
^₤^Baseline plasma albumin concentration (g/dL, mean (sd))		41.85 (4.87)	30.29 (4.63)	<0.001

Abbreviations: D4T = Stavudine, 3TC = Lamivudine, TNF = Tenofovir, EMT = Emtricitabine, AZT = Zidovudine. AIDS = Acquired Immuno-Deficiency Syndrome. HBV = Hepatitis B virus infection. ART = Antiretroviral therapy. ^¥^CYP3A5*1 represent functional wild type allele while *0 represent non-functional alleles which include *3, *5 and *7. ^‡^SLCO1B1*1b represent SNP rs2306283 (*SLCO1B1*.c388A > G) and SLCO1B1*5 represent SNP rs4149056 (*SLCO1B1*.c521T > C); Combination of the two SNPs form the following haplotypes: AT (*1a), AC (*5), GT (*1b), and GC (*15). ^₤^The baseline values were obtained before initiation of treatment.

### Population PK model parameters

**Figure 1:**
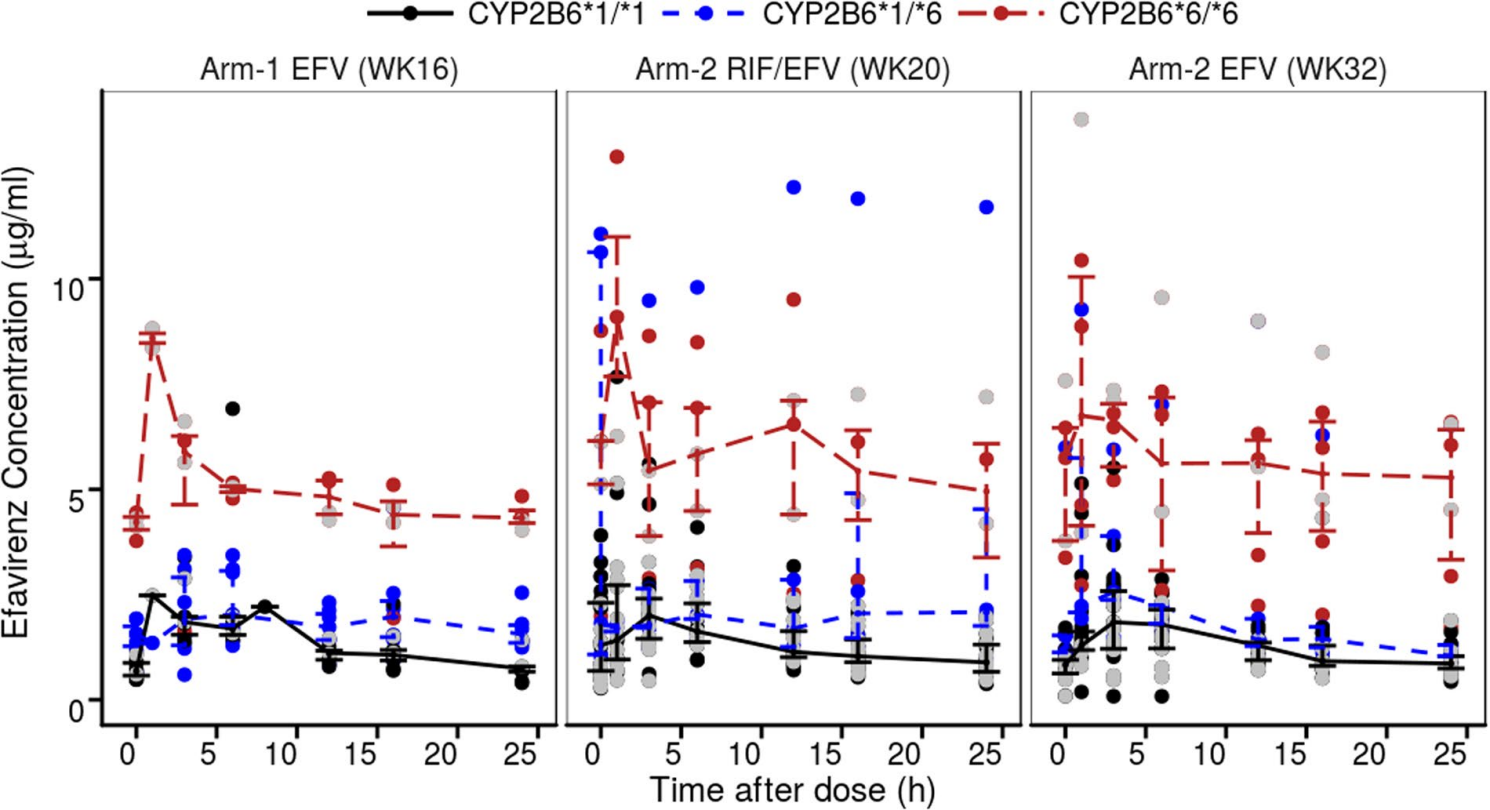
Observed efavirenz concentration vs time profiles for different *CYP2B6* genotypes at week 16 of EFV treatment in arm 1 and arm 2 and at week 32 (8 weeks after stopping co-treatment) in arm 2. The lines and error bars represent medians and 25^th^ to 75^th^ percentiles of the observed concentrations. The grey points are the observed concentrations for subjects with missing genotype data.

**Figure 2:**
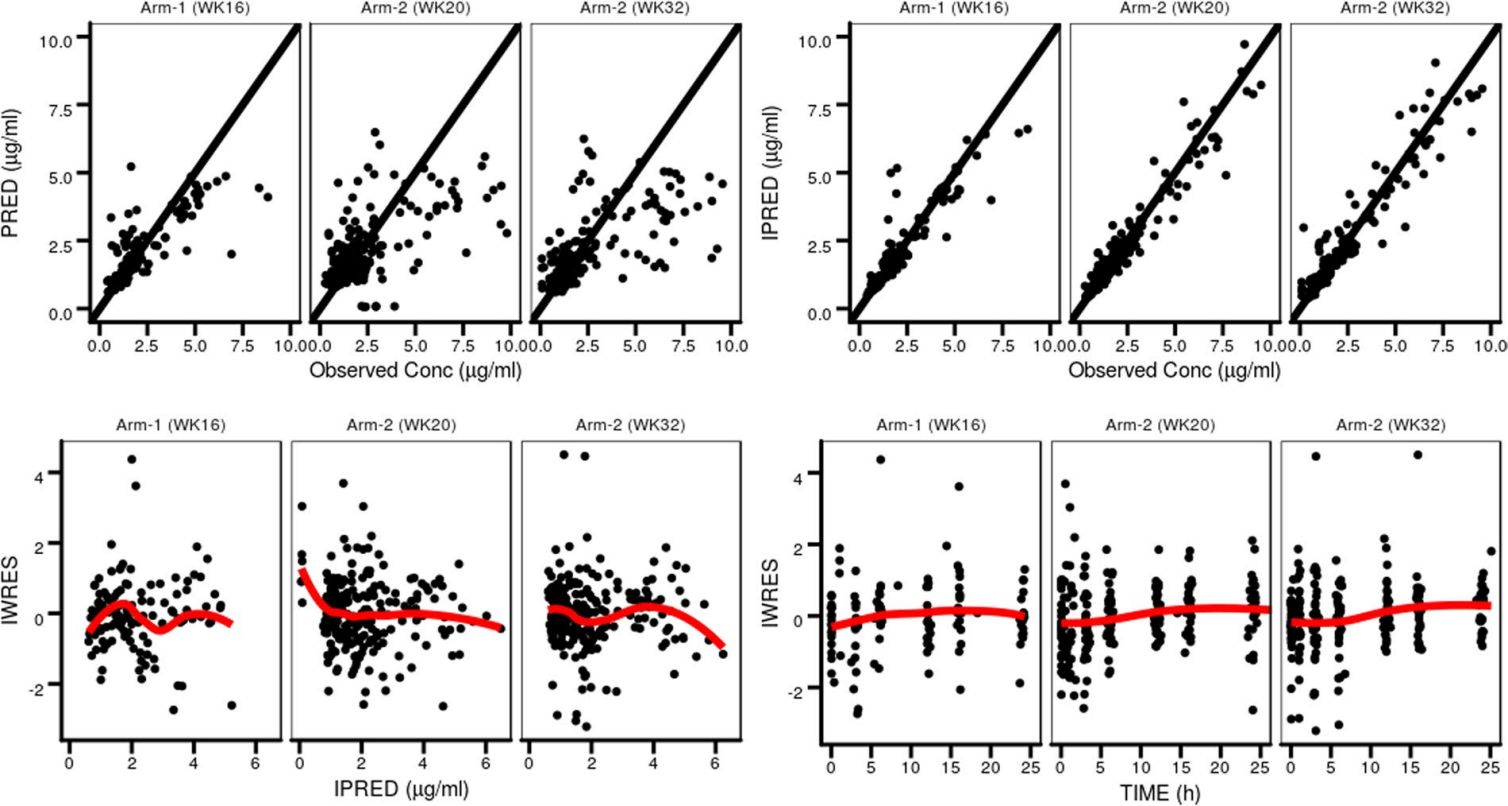
A goodness-of-fit plots for the final model. PRED = predicted population typical concentration, IPRED = predicted individual concentration, IWRES = Individual weighted residuals. Epsilon shrinkage was 11% and eta shrinkages were 11%, 12%, 39%, and 31 for between-subject variance of CL/F, V/F, KA, and Q/F. Eta shrinkage for between occasion variance of CL/F was at week 16 and 32 were 11% and 16 respectively.

**Figure 3:**
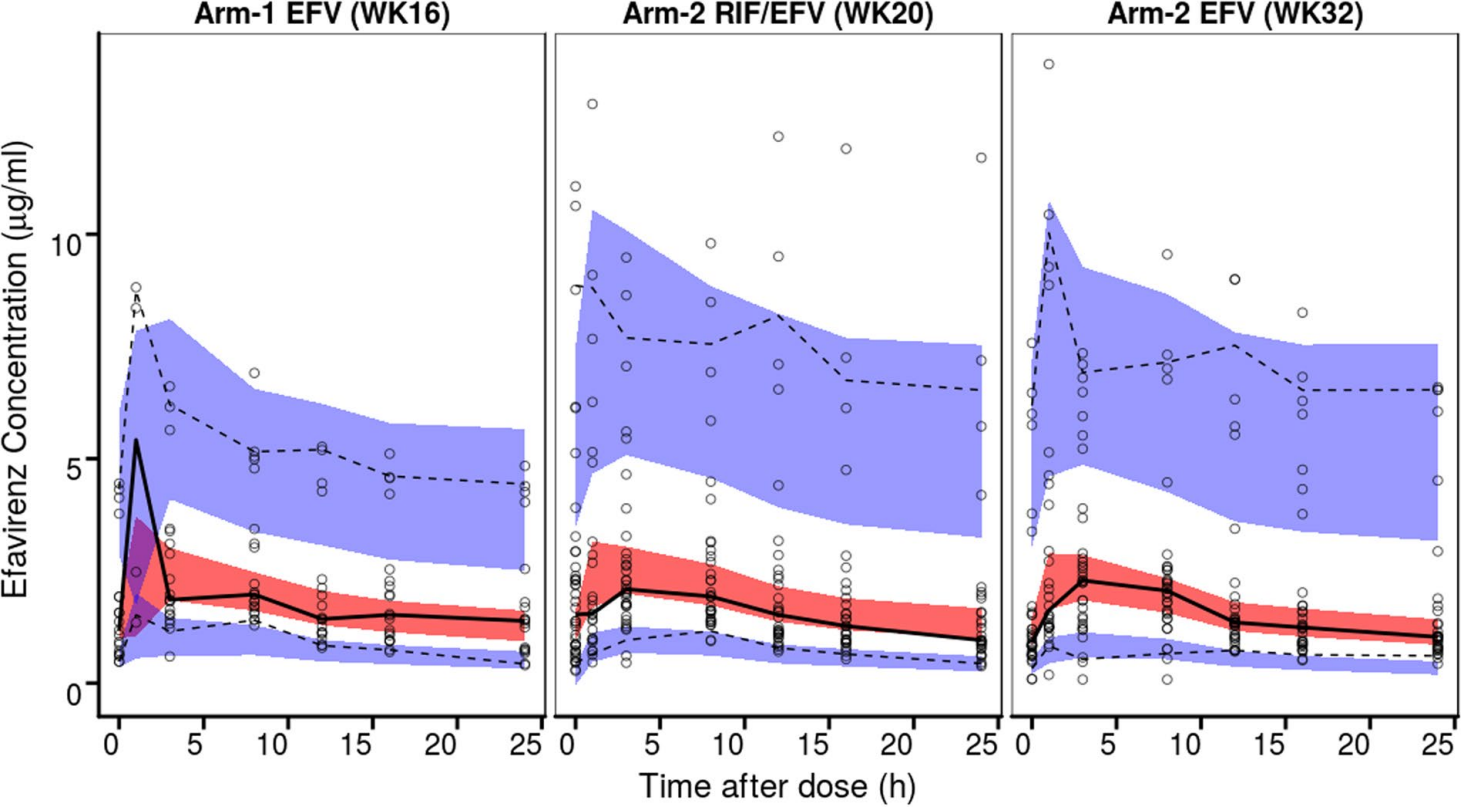
A visual predictive check of efavirenz concentration vs time profiles for arm 1 and arm 2 (Week 20 and 32). The open circles represent observed values, the solid line represents the median of the observed data, and the lower and upper dashed lines represent the 5th and 95th percentiles of the observed data. The red shaded area represents the 95% confidence interval of the medians of model prediction, while the lower and upper blue shaded areas represent the 95% confidence interval of the 5th and 95th percentiles of the model predictions.

The population-based pharmacokinetic analysis included all 556 efavirenz concentrations from 56 subjects. Figure 1 displays the concentration versus time profiles for efavirenz stratified by treatment arms, occasions and CYP2B6*6 genotypes. No concentrations were below the limit of quantification. Efavirenz pharmacokinetics was adequately described by a 2-compartment pharmacokinetic model, with first-order oral absorption kinetics, and without absorption lag (Supplementary Figure S2). A combined additive and proportional residual error model adequately described the unexplained variability in the concentration. The estimated population PK parameter values are as reported in Table 2. The table also reports the medians and 95% confidence intervals (computed by non-parametric bootstrap) which indicate a good precision the parameter estimates. Table 2 does not report the asymptotic relative standard errors (which assume asymptotic normality) because they were inestimable probably due to the small sample size^45^. For a typical subject in arm 1 with a CYP2B6*1/*1 genotype and weight of 70 kg, the estimated efavirenz clearance was 23.6 L/h. The estimated structural model parameter values for KA, V/F, Q/F and VP/F are 0.37 h^−1^, 314 L, 68, and 947 L, respectively, for a typical subject with a weight of 70 kg. Figure 2 shows the adequacy of the model fit through the goodness-of-fit plots. Figure 3 shows a good agreement between the model predictions and the observed data, indicating that the model can adequately be used to simulate steady-state efavirenz concentrations.

**Table 2:** Parameter estimates of the final population PK model.

Parameters	Description	Estimates	Bootstrap Median (95%CI)	Between Subject Variance	Bootstrap Median of Between Subject Variance (95%CI)	BSV (CV%)	Between Occasion Variance	Bootstrap Median of Between Subject Variance (95%CI)
$C{L}_{{1}_{1}}$	Typical clearance for CYP2B6*1/*1 in arm 1 (L/h)	23.4	23.7 (18–26.4)	0.104	0.096 (0.025–0.233)	33	0.0934925	0.0633 (0.0555–0.239)
V	Volume of distribution for central compartment (L)	209	198 (106–325)	0.91	0.954 (0.515–1.98)	122		
KA	Absorption rate constant (1/h)	0.344	0.318 (0.172–0.545)	0.412	0.331 (4.12e-05–0.929)	71.4		
Q	Intercompartmental clearance (L/h)	59.2	51.9 (37.1–104)	0.556	0.492 (5.56e-05–1.45)	86.3		
VP	Volume of distribution for peripheral compartment (L)	912	1288 (486–81570766)	0^a^	0^a^	0^a^		
ADD	Additive residual error (µg/L)	0.0873	0.0489 (0.000873–0.221)					
PROP	Proportional residual error	0.242	0.238 (0.207–0.292)					
${\Theta }_{{1}_{2}}$	Fraction of typical clearance for CYP2B6*1/*6 in arm 1	0.618	0.63 (0.396–0.899)					
${\Theta }_{{1}_{3}}$	Fraction of typical clearance for CYP2B6*6/*6 in arm 1	0.311	0.295 (0.273–0.591)					
${\theta }_{{21}_{1}}$	Fraction of clearance between arm 1 and arm 2 for CYP2B6*1/*1 during co-treatment	0.915	0.926 (0.759–1.11)					
${\theta }_{{21}_{2}}$	Fraction of clearance between arm 1 and arm 2 for CYP2B6*1/*6 during co-treatment	0.852	0.839 (0.48–1.6)					
${\theta }_{{21}_{3}}$	Fraction of clearance between arm 1 and arm 2 for CYP2B6*1/*6 during co-treatment	1.02	1.02 (0.694–1.68)					
${\varphi }_{{22}_{1}}$	Fraction of clearance between arm 1 and arm 2 for CYP2B6*1/*1 after co-treatment	1.19	1.15 (1–1.47)					
${\varphi }_{{22}_{2}}$	Fraction of clearance between arm 1 and arm 2 for CYP2B6*1/*6 after co-treatment	1.19	1.2 (0.86–1.55)					
${\varphi }_{{22}_{3}}$	Fraction of clearance between arm 1 and arm 2 for CYP2B6*1/*6 after co-treatment	1.05	1.05 (0.877–1.3)					
${\pi }_{\ast 1/\ast 1}$	Estimated proportion of CYP2B6*1/*1 among subjects with missing genotype	0.784	0.787 (0.561–0.967)					
${\delta }_{\ast 1/\ast 6}$	Estimated proportion of CYP2B6*1/*6 among subjects with missing genotype	0^a^	0^a^					
${\theta }_{SEX}$	Fraction of CL for male compared to female subjects	1.02	1.02(0.859–1.28)					

^a^Fixed to this value, not estimated. BSV = Between-subject variability (CV %).

### Population Mixture model for missing CYP2B6*6 genotype data

The mixture model estimated 78% and 22% of the subjects with missing genotype data to be the CYP2B6*1/*1 genotype. Consequently, the model identified 2 and 13 subjects among those with missing genotype data to be of the CYP2B6*1/*1 genotype in arm 1 and arm 2, respectively. The model also identified two subjects in arm 1 and two subjects in arm 2 to be of CYP2B6*6/*6 genotype.

### Influence of the covariates on the PK parameters

The CYP2B6*6 genotype was the only significant covariate of efavirenz clearance. Subjects with CYP2B6*1/*6 and CYP2B6*6/*6 in arm 1 had approximately 38% and 69% percent lower clearance, respectively, than their typical counterparts with the CYP2B6*1/*1 genotype. During anti-TB co-treatment, subjects in arm 2 had comparable efavirenz clearances as subjects in arm 1, irrespective of the CYP2B6*6 genotype status. For example, although typical clearance for arm 2 subjects with the CYP2B6*1/*1 genotype was 10% lower than that of subjects with a similar genotype in arm 1, this difference was not statistically significant. Similarly, although subjects with CYP2B6*1/*6 genotype in arm 2 had 15% lower clearance compared to subjects with a similar genotype in arm 1, the difference was not statistically significant.

After completion of anti-TB co-treatment, there was no statistically significant change in efavirenz clearance even eight weeks after stopping anti-TB therapy. For example, although efavirenz clearance increased by 19% in arm 2-CYP2B6*1/*1 genotype patients compared to clearance with co-treatment, this increase was not statistically significant. Similarly, efavirenz clearance increased insignificantly, by 5%, after co-treatment for subjects with CYP2B6*1/*6.

Sex had no impact on efavirenz clearance, as male subjects had comparable clearance (approximately 1.02 folds) to females.

## Discussion

The main finding of this study is that after 16 weeks of efavirenz treatment, clearance in subjects undergoing HIV treatment is comparable to that in patients undergoing anti-TB co-treatment. This finding supports the hypothesis that after long-term efavirenz treatment, the magnitude of autoinduction of metabolism and cellular transport is comparable to that due to efavirenz/rifampicin co-treatment. This hypothesis is also supported by several other studies, which compared efavirenz pharmacokinetics after at least 12 weeks of treatment when taken with or without rifampicin-based tuberculosis co-treatment. A study with similar design and comparable sample size as this study (twin study), but in Ethiopian subjects, had similar findings^31^. Similarly, *Ramachandran et al*. found comparable efavirenz pharmacokinetics with and without anti-TB co-treatment in a study that assessed efavirenz PK after a mean treatment duration of 18 weeks among HIV-infected subjects only^46^. In a study among Zimbabwean subjects, efavirenz PK was assessed after a mean duration of 6 months among subjects receiving HIV treatment and anti-TB co-treatment. The findings of this study indicated comparable efavirenz PK in both groups^43^. A study among South African subjects that assessed efavirenz PK after at least 14 weeks of treatment in HIV-infected subjects also found comparable efavirenz PK^47^. In a parallel comparative study where plasma EFV concentration was monitored up to 8 months, *Mukonzo et al*. reported that rifampicin-based anti-TB co-treatment reduces efavirenz plasma exposure during early therapy (2 weeks) but has no significant long-term effect in Ugandan patients^48^.

Compared with single dose EFV clearance reported elsewhere, the estimated clearance in this study is considerably higher and this is consistent with EFV autoinduction properties. For subjects with CYP2B6*1*1 genotype, single dose EFV clearance have been reported to range between 4–7.5 L/h^49–51^ while after multiple dosing EFV clearance is reported to range between 9.4–15.5 L/h^26,31,52–54^ (Estimates obtained without normalizing clearance by body weight). Upon normalizing EFV clearance by body weight Matteeli *et al*. reported EFV clearance of 18.2 L/h^55^ for a 70 kg person which is comparable to the value estimated in this study of 23 L/h for a 70 kg person.

To our knowledge, no long-term efavirenz treatment study has reported lower efavirenz exposure (indicating higher clearance or low bioavailability) among subjects on anti-TB co-treatment compared to subjects on efavirenz without rifampicin co-treatment. In contrast, several studies have reported higher exposure during anti-TB co-treatment than during efavirenz-only treatment^20,21,37–40^. The practical implication of this finding is that rifampicin co-treatment should not be a criterion for efavirenz dose adjustment among subjects undergoing anti-TB co-treatment. However, on the other hand, efavirenz exposure among subjects on long-term efavirenz treatment (after at least 16 weeks) should be evaluated to identify and appropriately manage subjects with levels below the therapeutic range and therefore increased the risk of virological failure.

Our study also finds that after completion of anti-TB co-treatment, efavirenz clearance does not significantly increase among subjects with functional and dysfunctional *CYP2B6* enzymes. Although the increase in this study is not significant, our finding supports several other previous reports that have reported comparable or significantly lower efavirenz exposure or higher efavirenz clearance after completion of anti-TB co-treatment compared to during anti-TB co-treatment. In a twin study among Ethiopian subjects, the measures of efavirenz exposures (AUC_0–24_, Cmax, and Cmin) were comparable during and after completion of efavirenz co-treatment^31^. Similarly, a study among Tanzanian subjects by *Semvua et al*. found comparable exposures^32^. However, a study by *Uttayamakul et al*. reported significantly lower mid-dose efavirenz concentration and a study by *Gengiah et al*. reported higher efavirenz clearance after completion of anti-TB co-treatment^30,57^. Taken together, these findings indicate that efavirenz clearance may or may not increase after completion of rifampicin treatment. The likely explanation for this observation comes from the involvement of the N-acetyl transferase-2 (*NAT2*) enzyme in modifying efavirenz drug-drug interactions. The *NAT2* enzyme metabolizes isoniazid, an anti-tuberculosis drug co-administered with rifampicin for the six months of tuberculosis treatment. *NAT2* genetic variation results in 3 sub-population of isoniazid acetylators: rapid, intermediate, and slow acetylators, with relatively low, intermediate and high isoniazid exposure, respectively. Isoniazid inhibits *CYP2A6*, a liver enzyme responsible for approximately 23% of efavirenz metabolism^59^. Therefore, the *NAT2* polymorphism may cause differential inhibition of the *CYP2A6* enzyme by isoniazid and hence the inter-individual variation in the effect of anti-TB co-treatment on efavirenz PK. As a result, depending on *CYP2B6* genotype status, the completion of anti-TB co-treatment among rapid acetylators (with relatively low isoniazid exposure and therefore less inhibition) may be associated with non-significant changes or decreases in efavirenz clearance (as result of alleviation of RIF induction). In contrast, for slow acetylators, the completion of anti-TB co-treatment may be associated with an increase in efavirenz clearance (as a result of alleviation of isoniazid inhibition). This influence of *NAT2* polymorphism on the change of efavirenz clearance after completion of anti-TB co-treatment has been reported previously^26,60^. In the study by *Bertrand et al*., efavirenz clearance decreased by 24% after completion of anti-TB co-treatment among rapid acetylator subjects with the CYP2B6*1/*1 genotype, but increased by 11.6%, 33% and 19% among slow acetylators with CYP2B6*1/*1, *1/*6 and *6/*6 genotype, respectively. Unfortunately, direct comparisons of the clearances before and after completion of co-treatment were not reported, and therefore it is difficult to comment on the statistical significance of the change^26^.

Our analysis identified the CYP2B6*6 genotype to be a major determinant of the magnitude of efavirenz clearance. Subjects without the mutation (homozygous wild type) had the highest typical efavirenz clearance followed by subjects with one mutation (heterozygous), while subjects with double mutations (homozygous mutated) had the least clearance. This finding was expected, as many previous studies have identified *CYP2B6* as the main efavirenz metabolizing enzyme and non-synonymous mutations, particularly CYP2B6*6 c.*516 G > T*, result in a protein with dysfunctional enzyme activity^61,62^. Our findings of approximately 40% and 70% reduction of efavirenz clearance in subjects with CYP2B6*1/*6 and *6/*6 genotypes, respectively, are comparable to the relative difference in efavirenz clearance values reported by *Bertrand et al*. among subjects receiving HIV treatment alone^26^. Similar reductions in efavirenz clearance by CYP2B6*6 genotypes have been reported by *Hui et al*.^52^. However, other magnitudes of reductions have been reported in other studies. *Cabrera et al*. reported 50% and 75% reductions (under steady-state conditions) in HIV subjects with CYP2B6*1/*6 and *6/*6 genotypes, respectively, while *Robarge et al*. reported 25% and 51% reductions among healthy subjects with CYP2B6*1/*6 and *6/*6 genotypes, respectively, in a single-dose efavirenz pharmacokinetic-pharmacogenetic study^49,53^. Similarly, in a single-dose pharmacokinetic study among healthy volunteers, *Mukonzo et al*. reported only a 21% reduction in clearance among subjects with the CYP2B6*6/*6 genotype but reported 20% and 55% reductions in CYP2B6*1/*6 and *6/*6 genotypes, respectively, among HIV-infected subjects who completed at least 14 days of efavirenz treatment^14,50^. Taken together, these findings imply that the CYP2B6*6 genotype is important for planning genotype-based dosing of efavirenz; however, estimation of efavirenz doses for the different genotypes should consider their relative influences on efavirenz pharmacokinetics after long-term treatment. Similar *CYP2B6* genotype-based efavirenz dose recommendations regardless of rifampicin-based anti-tuberculosis co-treatment for sub-Saharan Africa population have been published recently^14,63^.

In our analysis, sex was not an important covariate for efavirenz PK. However, sex has been identified by previous studies as an important covariate for efavirenz clearance. Studies by *Nyakutira et al*. and *Nemaura et al*. indicated higher efavirenz clearance in males than females^31,32^, while a study by *Dhoro et al*. indicated the opposite^43^. *Lamba et al*. indicated higher *CYP2B6* transcription and expression in hepatocytes obtained from human livers of females compared to male organ donors^64^. Therefore, to control for the possible confounding effect of sex in this analysis, a full covariate model building approach was taken, and we included sex as a covariate of clearance.

In this analysis, we did not control for week 16 albumin levels. Several studies have shown lower than normal albumin levels during chronic infection. *Kuteesa et al*. reported lower albumin levels in HIV/TB co-infected subjects than in HIV-only subjects before antiretroviral treatment (ART). However, after treatment, the albumin level increased, and the steady state rate of albumin production in both arms was comparable^65^. Based on these previous findings, albumin levels were not expected to differ between HIV-only and HIV-TB patients after 16 weeks of ART. Therefore, we did not consider the need to control for albumin levels in this analysis.

One of the limitations of our study was that the two treatment arms were not comparable in demographic, social and baseline clinical characteristics. This is because assignment to the treatment arms was by disease status (HIV versus HIV/TB coinfection) and matching of subjects by characteristics was not possible for ethical reasons. However, we tried to attenuate the effects of confounders by including all the reported covariates of efavirenz PK into the final efavirenz PK covariate model. Sex and weight were the only previously identified covariates whose data were available during the analysis. Therefore, efavirenz clearance was allometrically scaled by weight and sex and was included as a covariate of clearance. Another limitation was that genotype information was missing for approximately 35% of the recruited subjects. This limitation was controlled by using population mixture modeling to impute the genotype data based on the measured efavirenz concentration. Compared to sub-population 1 of the mixture model, ETA shrinkage for subpopulation 3 was high (Supplementary Table 2), reflecting a small deviation of individual parameters from the typical parameter value of the sub-population. This is expected because of the small number of subjects in this subpopulation. None of the subjects with missing CYP2B6*6 genotype data were identified in subpopulation 2, and therefore ETA shrinkage for this subpopulation was 100%.

In conclusion, CYP2B6*6 genetic polymorphism, but not rifampicin co-treatment, should be considered when adjusting for efavirenz dosage during both ART and anti-TB co-treatment. Furthermore, for planning *CYP2B6* genotype-based dosing of efavirenz, the impact of the polymorphism on efavirenz exposure should be assessed after at least 16 weeks of efavirenz treatment to achieve complete autoinduction. However, considering the inter-individual variations in the effect of anti-TB co-treatment on efavirenz PK (rifampicin induction and isoniazid inhibition), it might be advisable to use therapeutic drug monitoring to initially adjust EFV doses.

## Electronic supplementary material

Supplementary Information

## Data Availability

The dataset analysed during the current study are available from the corresponding author on reasonable request.
