# Supplementary material for: Long-term efavirenz pharmacokinetics is comparable between Tanzanian HIV and HIV/Tuberculosis patients with the same CYP2B6*6 genotype
Source: Sci Rep. 2018 Nov 5;8:16316. doi: 10.1038/s41598-018-34674-3 (PMC6218524; doi:10.1038/s41598-018-34674-3)
Supplement: Supplementary file 1 — Supplementary Information [file 41598_2018_34674_MOESM1_ESM.pdf]

Long-term efavirenz pharmacokinetics is comparable between Tanzanian HIV and HIV/Tuberculosis patients with the same *CYP2B6*6* genotype

*Eliford Ngaimisi Kitabi, [1,2] Omary Mashiku Sylivester Minzi,[1] Sabina Mugusi,[3] Philip Sasi,[3] Mohamed Janabi, [5]Ferdinand Mugusi,[5] Leif Bertilsson,[2] Jürgen Burhenne[4], Eleni Aklillu[2].

NONMEM CONTROL STREAM FOR THE FINAL MODEL

```
$PROBLEM    EFAVIRENZ FINAL MODEL
$INPUT  ID TIME TAD TADBIN DV AMT MDV EVID II SS ARM OCC OCCINTV
        AGE SEX WT BMI ARV SMOK ALC SHIN HBV VDRL CP2B CP3A SLCO
        GROUP STRATA
$DATA     ../data/efvipk2017sep28.csv IGNORE=@
$SUBROUTINE ADVAN6 TOL=6
$MODEL     NCOMPS=3 COMP=(GUT1) COMP=(CENT,DEFOBS) COMP=(PERI,NODOSE)
;;;Use population mixture model to impute missing CYP2B6 genotype data
$MIX
NSPOP=3        ; Specify 3 subpopulation in the missing data
P(1) = THETA(16)
P(2) = (1 - P(1)) * THETA(17)
P(3) = 1 - P(1) - P(2)
$PK
EST = MIXEST
;;; Create dummy variable for sex
MALE=0
IF(SEX.EQ.2) MALE=1
;;; Create dummy variable for available and imputed genotype data
CP2BMIXNUM = 1
IF(CP2B.EQ.2) CP2BMIXNUM = 2
IF(CP2B.EQ.3) CP2BMIXNUM = 3
IF(CP2B.EQ.-99.AND.MIXNUM.EQ.1) CP2BMIXNUM = 1
IF(CP2B.EQ.-99.AND.MIXNUM.EQ.2) CP2BMIXNUM = 2
IF(CP2B.EQ.-99.AND.MIXNUM.EQ.3) CP2BMIXNUM = 3
;;; Create dummy variable for CYP2B6*6 genotype to use for estimation
;;;;of fold difference in efavirenz clearance between arm 1 and arm 2 and between arm 2 occassions.
B1 = 0
B2 = 0
B3 = 0
IF(CP2BMIXNUM.EQ.1) B1=1
IF(CP2BMIXNUM.EQ.2) B2=1
IF(CP2BMIXNUM.EQ.3) B3=1
;;;Create dummy variable for arm-2
A2 = 0
IF(ARM.EQ.2) A2=1
;;;Create dummy variable for Arm-2 and occasion 2
O2 = 0
IF(OCC.EQ.2) O2=1
;;; Define typical subpopulation clearances for MU modeling
LTVCL=LOG(THETA(1))+B2*LOG(THETA(8))+B3*LOG(THETA(9))+A2*(B1*LOG(THETA(10))+B2*LOG(
THETA(11))+B3*LOG(THETA(12)))+O2*(B1*LOG(THETA(13))+B2*LOG(THETA(14))+B3*LOG(THETA(15
)))+0.75*(LOG(WT)-LOG(70))+ MALE*LOG(THETA(18))
 ;;;Define typical subpopulation apparent volume of distribution
LTVV  = LOG(THETA(2))+ (LOG(WT)-LOG(70))
TVKA = THETA(3)
TVQ = THETA(4)
```

```
TVVP = THETA(5)
;;;Define MU models
MU_1 = LTVCL
MU_2 = LTVV
MU_3 = LOG(TVKA)
MU_4 = LOG(TVQ)+0.75*(LOG(WT)-LOG(70))
MU_5 = LOG(TVVP)+(LOG(WT)-LOG(70))
;;;Define between occasion variability for clearance
BOVCL=1
IF(GROUP.EQ.21) BOVCL=DEXP(ETA(6))
IF(GROUP.EQ.22) BOVCL=DEXP(ETA(7))
;;;Define stochastic models
CL = DEXP(MU_1+ETA(1))*BOVCL
V  = DEXP(MU_2+ETA(2))
KA = DEXP(MU_3+ETA(3))
Q = DEXP(MU_4+ETA(4))
VP = DEXP(MU_5+ETA(5))
;; First order rate constants (h-1)
S2   = V                                    ; scale for CENTRAL compartment
KA   = KA                                   ; Absorption rate constant
K    = CL/V                                 ; Elimination rate constant
K23  =Q/V
K32  =Q/VP
;;;Define differential equations for 2 compartment model
$DES
;;;Define rates
INPUT1=A(1)*KA
TOPERI=A(2)*K23
FROMPERI=A(3)*K32
OUTPUT2=A(2)*K
;;;Define differentials
DADT(1) = -INPUT1
DADT(2) = INPUT1+FROMPERI-OUTPUT2-TOPERI
DADT(3) = TOPERI-FROMPERI

$ERROR
IPRED = F
IRES = DV-IPRED
ADD=THETA(6)
PROP=THETA(7)*IPRED
W=SQRT(ADD*ADD+PROP*PROP)
IWRES=IRES/W
Y= IPRED + W*ERR(1)                    ; Model prediction of observed PK value with additive + proportional error

;; Initial estimates Theta and Omega
$THETA
 (0,23.3545)          ; CL
 (0,208.817)          ; V
 (0,0.344479)         ; KA
 (0,59.1631)          ; Q
 (0,912.068)          ; VP
 (0,0.0872526)        ; ADD
 (0,0.241611)        ; PROP
 (0,0.618486)         ; CLCP2B2
 (0,0.310718)         ; CLCP2B3
 (0,0.914908)         ; ARM2OCC1CP2B1
```

```
 (0,0.851619)        ; ARM2OCC1CP2B2
 (0,1.01737)         ; ARM2OCC1CP2B3
 (0,1.18644)         ; ARM2OCC2CP2B1
 (0,1.19174)        ; ARM2OCC2CP2B2
 (0,1.04729)         ; ARM2OCC2CP2B3
 (0,0.783883,1)    ; PROPCP2B1
 0 FIX                ; PROPCP2B2
 (0,1.01959,5.00)               ; CLMALE
$OMEGA  BLOCK(2)
 0.103587                        ;      BSVCL
 0.300906 0.910193            ;       BSVV
$OMEGA  BLOCK(1)  0.411878     ;      BSVKA
$OMEGA  BLOCK(1)  0.556338     ;       BSVQ
$OMEGA  BLOCK(1) FIX 0           ;      BSVVP
$OMEGA  BLOCK(1) 0.093522      ;    BOVCL21
$OMEGA  BLOCK(1) SAME         ;      BOVCL22
$SIGMA  1  FIX                            ; Scaled RUV variance

$ESTIMATION METH=1 INTER MAXEVALS=9999 PRINT=5 NOHABORT SIG=3 MSFO=msf10

$COVARIANCE

$TABLE      ID TIME TAD TADBIN EVID IPRED IWRES CWRES NPDE CL V KA Q
            VP ETA1 ETA2 ETA3 ETA4 ETA5 ETA6 SEX SMOK OCC GROUP CP2B
            ARV ALC SHIN HBV VDRL CP2B CP3A SLCO WT AGE BMI EST
            NOPRINT ONEHEADER FILE=mytab017
```

Supplementary Table S1: Parameters, objective functions and convergence status of the tested models during population PK model development

Parameters	run01	run02	run03	run04	run05	run06	run07	run08	run09	run010	run011	run012	run013	run014	run015	run016	run017
	1 CMT	2CMT	2CMT DUAL ABS	2CMT BSVCL	2CMT BSV(CL,V, KA)	2CMT BSV(CL,V, KA, Q)	2CMT BSV(CL,V, KA, VP)	2CMT BSV(CL,V, KA, Q,VP)	2CMT BSV(CL,V, KA) BOV(CL)	2CMT BSV(CL,V, KA,Q) BOV(CL)	2CMT BSV(CL,V, KA,VP) BOV(CL)	2CMT BSV(CL,V, KA,Q) BOV(CL)	2CMT BSV(CL,V, KA,Q) BOV(CL) COV(CL,V)	2CMT BSV(CL,V, KA,Q) BOV(CL) COV(CL,V, KA)	2CMT BSV(CL,V, KA,Q) BOV(CL) COV (CL,V, Q)	NO IMPUTE Missing CYP2B6	IMPUTE CYP2B6 FINAL MODEL
CL	10.668	10.723	7.329	13.377	13.648	13.755	13.656	13.775	13.649	13.732	13.684	13.731	13.702	13.674	13.69	18.816	23
V	386.487	170.168	3.468	389.432	275.024	314.348	267.165	291.401	186.987	208.366	191.252	208.439	106.978	165.351	117.838	210.704	208.7
KA	6.219	1.393	0.761	0.91	0.466	0.555	0.446	0.503	0.312	0.391	0.312	0.391	0.294	0.458	0.319	0.327	0.344
Q		219.859	172.695	27.029	32.814	29.481	34.398	34.067	42.479	44.286	41.283	44.292	51.97	56.437	52.783	48.821	59.1
VP		5506.12	7008.84	36442900000	505457000	276632000	7750.88	1937.37	927.57	713.791	1079.17	713.75	604.015	531.487	596.015	1479.01	912
D2			2.922														
F1			0.322														
ALAG1			0.179														
ALAG2			0.926														
ADD	1.784	0.01	0.01	0 [a]	0 [a]	0 [a]	0 [a]	0 [a]	0.044	0.095	0.034	0.095	0.084	0.083	0.082	0.106	0.087
PROP	0.556	0.916	0.311	0.352	0.305	0.298	0.304	0.296	0.265	0.239	0.266	0.239	0.242	0.241	0.242	0.235	0.242
BSVCL	0 [a]	0 [a]	0 [a]	0.35	0.362	0.361	0.363	0.364	0.346	0.345	0.345	0.345	0.343	0.342	0.341	0.263	0.104
BSVV	0 [a]	0 [a]	0 [a]	0 [a]	0.185	0.355	0.223	0.431	0.491	0.505	0.488	0.505	0.94	1.058	0.893	1.196	0.91
BSVKA	0 [a]	0 [a]	0 [a]	0 [a]	1.197	0.959	1.112	0.886	0.531	0.797	0.525	0.797	0.398	1.245	0.41	0.372	0.412
BSVQ		0 [a]	0 [a]	0 [a]	0 [a]	0.409	0 [a]	0.482	0 [a]	0.563	0 [a]	0.563	0.608	0.788	0.67	0.473	0.556
BSVVP		0 [a]	0 [a]	0 [a]	0 [a]	0 [a]	11.685	4.532	0 [a]	0 [a]	0.395	0 [a]	0 [a]	0 [a]	0 [a]	0 [a]	0 [a]
BSVD2			0 [a]														
BSVF1			0 [a]														
BSVALAG1			0 [a]														
BSVALAG2			0 [a]														
BOVCL21									0.074	0.082	0.067	0.082	0.085	0.086	0.087	0.069	0.093
BOVCL22									0.074	0.082	0.067	0.082	0.085	0.086	0.087	0.069	0.093
CLCP2B2																0.67	0.618
CLCP2B3																0.275	0.311
ARM2OCC1CP2B1																1.141	0.915

ARM2OCC1CP2B2																0.893	0.852
ARM2OCC1CP2B3																1.546	1.017
ARM2OCC2CP2B1																1.194	1.186
ARM2OCC2CP2B2																1.243	1.192
ARM2OCC2CP2B3																1.144	1.047
CLMALE																1.12	1.02
PROPCP2B1																	0.784
PROPCP2B2																	0
OFV	1472	1451.083	1459.557	284.704	190.519	191.425	191.806	189.83	114.251	96.614	113.701	96.614	81.9	78.819	80.665	50.305	41.367
**STATUS**	**1**	**1**	**0**	**0**	**1**	**1**	**1**	**1**	**1**	**1**	**1**	**1**	**1**	**0**	**0**	**1**	**1**

a = Fixed to this value, Not estimated. BO = between occasion variance. BSV = between subject variability (VARIANCE VALUES), COV = variance-covariance matrix, STATUS = (1 = minimization successful, 0 = minimization terminated). Base model = run013, Final model = run017. Full covariate model had reduced BSV of clearance by about 51% (final model compared to base model).

Supplementary Table S2: ETA and EPS shrinkage for 3 subpopulations identified by mixture modeling

Parameter	Description	Submodel 1	Submodel 2	Submodel 3
ETA1	ETA shrinkage for CL (%)	11	100	51
ETA2	ETA shrinkage for V (%)	12	100	52
ETA3	ETA shrinkage for KA (%)	39	100	50
ETA4	ETA shrinkage for Q (%)	31	100	68
ETA5	ETA shrinkage for VP (%)	100	100	100
ETA6	ETA shrinkage for BOVCL occasion 1 (%)	23	100	94
ETA7	ETA shrinkage for BOVCL occasion 2 (%)	44	100	66
EPS	Epsilon shrinkage (%)	11	100	33

Supplementary Figures

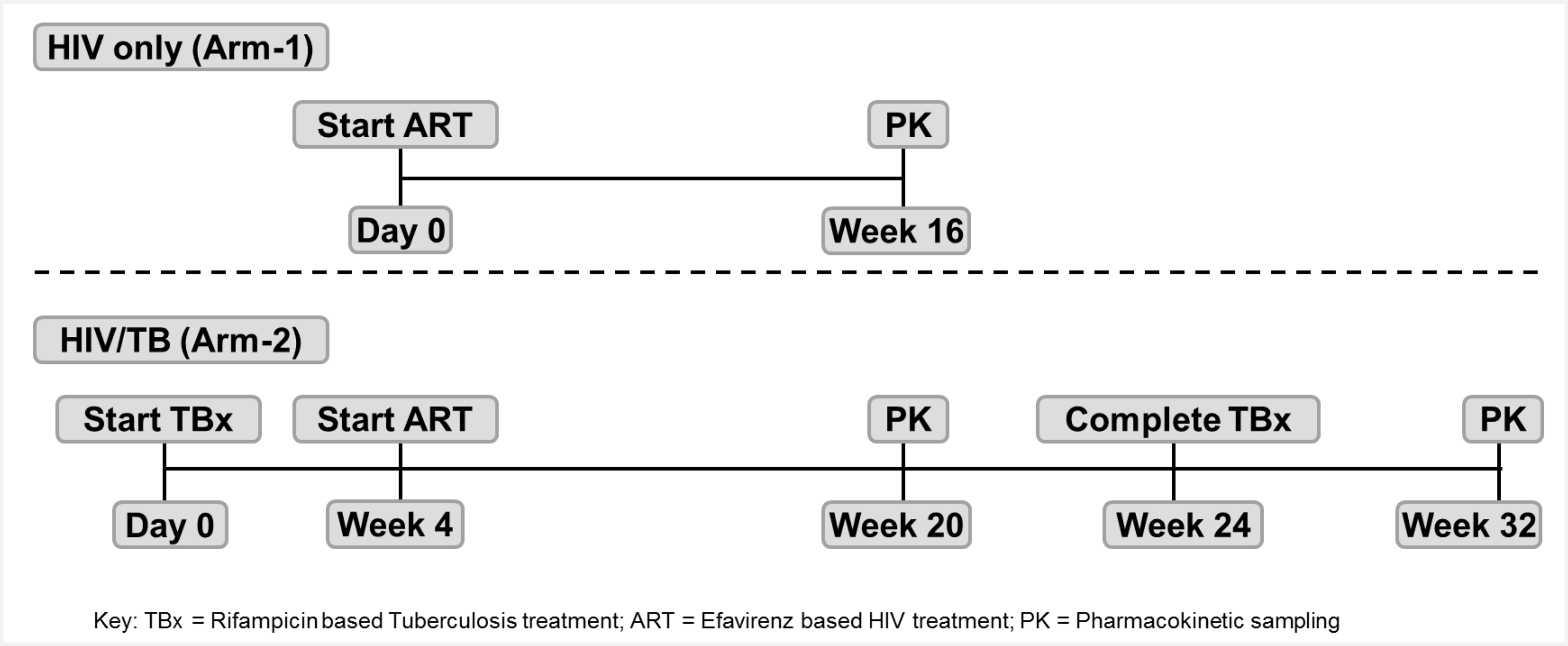

Figure S1. A schematic representation of the study design

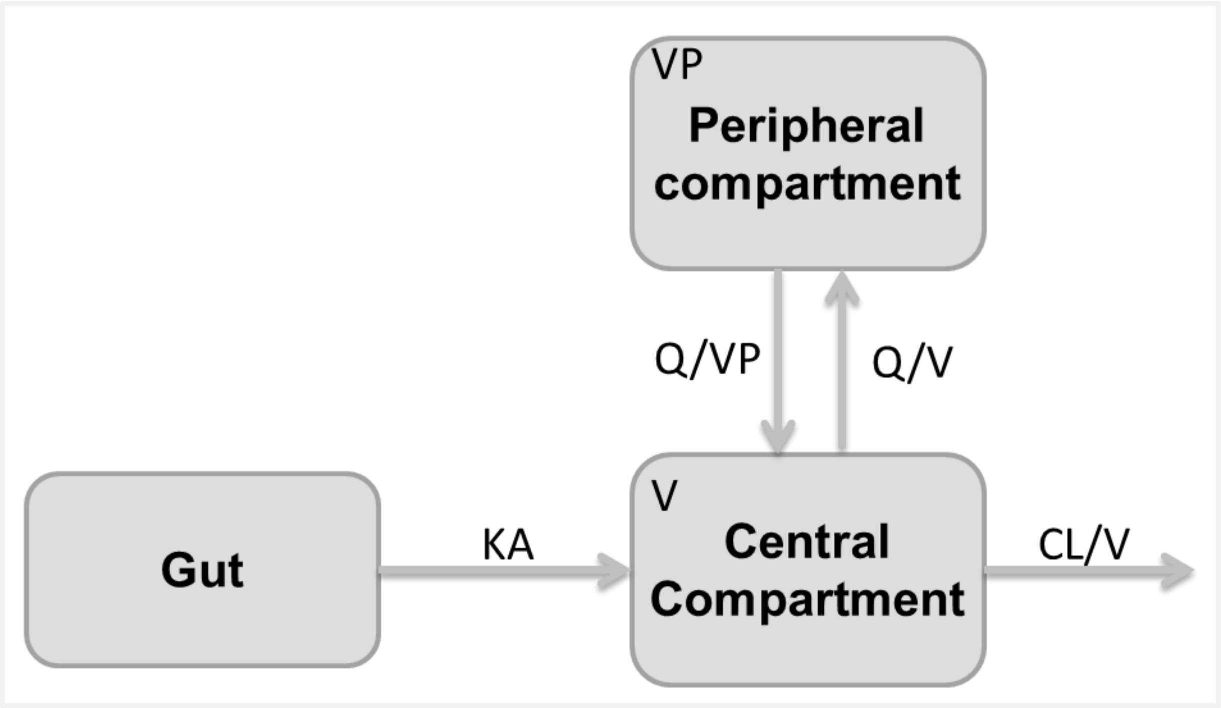

Figure S2 A schematic illustration of the 2 compartment pharmacokinetic model for efavirenz. KA = absorption rate constant from the gut; CL = clearance from central compartment; Q = intercompartment clearance; V= volume of distribution of the central compartment; VP = Volume of distribution of the peripheral compartment; CL/V = rate constant of elimination from the central compartment; Q/V rate constant of transfer from central compartment to peripheral compartment; Q/VP = rate constant of transfer from peripheral to central compartment.
